# Clinical Evaluation of a Novel Stool Antigen Test Using Bioluminescent Enzyme Immunoassay for Detecting *Helicobacter pylori*

**DOI:** 10.1155/2022/5571542

**Published:** 2022-04-21

**Authors:** Toshihiko Kakiuchi, Muneaki Matsuo, Yasuhisa Sakata, Kazuma Fujimoto

**Affiliations:** ^1^Department of Pediatrics, Faculty of Medicine, Saga University, Saga 849-8501, Japan; ^2^Department of Internal Medicine, Faculty of Medicine, Saga University, Saga 849-8501, Japan; ^3^Department of Gastroenterology, International University of Health and Welfare, Ohkawa, Fukuoka 831-0016, Japan

## Abstract

**Background:**

BLEIA ™ “EIKEN” *Helicobacter pylori* antigen (B[EIA]) is based on the bioluminescent enzyme immunoassay (BLEIA) method that was newly developed with high sensitivity in detecting *Helicobacter pylori* (*H. pylori*) antigen in feces.

**Methods:**

In the project for *H. pylori* screening and treatment in Saga Prefecture in 2019, 141 students received the stool *H. pylori* antigen test as a secondary test. For 141 students, a comparative test was conducted between B (EIA) and extracorporeal diagnostic agents that were marketed in Japan as of 2019. The detection performance of *H. pylori* ATCC43504 standard strain and *H. pylori* antigen in commercial human fecal specimens were conducted.

**Results:**

The comparison of B (EIA) with Quick Chaser ^TM^*H. pylori* (Q [IC]) revealed positive and negative concordance ratios of B (EIA) to Q (IC) of 100.0% (110/110) and 71.0% (22/31), respectively. A comparative test was conducted between B (EIA) and extracorporeal diagnostic agents that were marketed in Japan as of 2019, and B (EIA) was most sensitive on “detecting *H. pylori* antigen of ATCC43504 standard strain” and “detecting *H. pylori* antigen in commercial human fecal specimens,” compared with other kits. Nine dissociated specimens that were negative for Q (IC) and positive for B (EIA) were confirmed. The measured value of B (EIA) in the dissociation samples were 1.3–87.4 cutoff index in the range that can be evaluated as negative by other fecal *H. pylori* antigen test kits, all the dissociation samples were *H. pylori* antigen-positive cases, and finally the cause of result divergence was presumed as false negative due to insufficient sensitivity of Q (IC).

**Conclusion:**

B (EIA) that is based on the BLEIA method, which applies firefly luciferase luminescence, is more sensitive than stool antigen test kits that are currently marketed in Japan and is very useful in diagnosing *H. pylori* infection, especially in situations where noninvasive tests are preferred, such as in children.

## 1. Introduction

One of the major risk factors for gastric cancer is *Helicobacter pylori* (*H. pylori*) infection [1–4]. The risk of stomach cancer among individuals who are not infected with *H. pylori* is extremely low [5, 6], and its risk is lowered by the eradication therapy for *H. pylori*. In addition, preventing gastric cancer is more possible during the early stage of bacterial infection [7–10].

In Japan, the gastric cancer mortality rate is second highest in males and third highest in females among all cancer mortalities [11]. Recently, the number of municipalities in Japan that conduct *H. pylori* testing and eradication in middle school students to prevent gastric cancer has been increasing [12, 13]. In 2016, Saga Prefecture became one of the first prefectures in Japan to begin *H. pylori* testing in all third-year middle school students and eradication therapy for those who tested positive [14]. *H. pylori* tests that were conducted throughout Japan differ across municipalities; many regions use the urine *H. pylori* antibody test as the primary test and then use either the urea breath test or the stool *H. pylori* antigen test as a secondary test [15–17]. A kidney disease screening program was established for school-age children in Japan; thus, urine is used for the primary test. The fecal antigen test has been used as an examination method for *H. pylori* infection as the secondary test, thus its high inspection accuracy is required compared with primary test methods.

BLEIA ™ “EIKEN” *H. pylori* antigen (B[EIA]; Eiken chemical CO., LTD., Tokyo, Japan) that is based on the bioluminescent enzyme immunoassay (BLEIA) method [18] was newly developed for detecting *H. pylori* antigen in feces with high sensitivity. B (EIA) applies firefly luciferase luminescence, which is a type of bioluminescence, wherein the substrate luciferin is converted to oxyluciferin by the firefly luciferase in the presence of adenosine triphosphate, magnesium ion, and dissolved oxygen, thus light emission is obtained. The enzyme stability has improved using heat-resistant biotinylated luciferase [19, 20].

This study aimed to clinically evaluate the efficacy of B (EIA) for school-age children and compare it with that of the already commercially available rapid test kits.

## 2. Methods

### 2.1. Ethical Approval and Consent to Participate

The institutional review board of Saga University Hospital approved the present study (approval numbers: 2019-04-04). The study methodology was explained before obtaining written informed consent from all participants and their parents or guardians.

### 2.2. Enrollment

**Figure 1:**
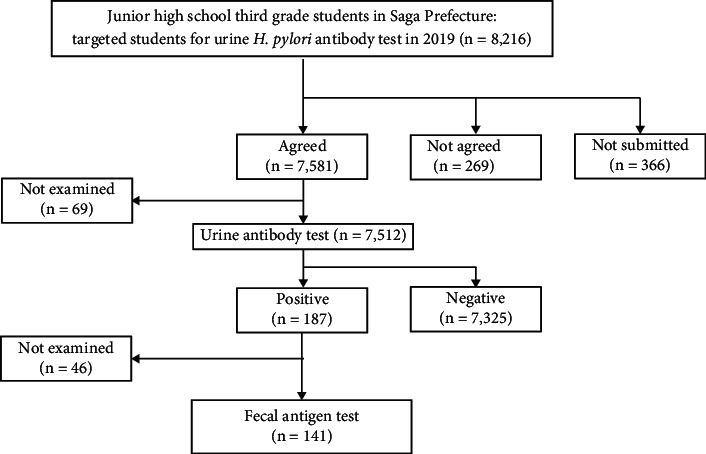
Flowchart of participants in the present study. Of the 187 positive urinary *H. pylori* antibody tests, 141 were included in the present study.

The longitudinal project for *H. pylori* screening and treatment among junior high school third-grade students in Saga Prefecture started in 2016, which aimed to prevent primary gastric cancer [14].  Figure 1 shows a flowchart of the junior high school third-grade students in Saga Prefecture in 2019. Among 8,216 junior high school students aged 14 or 15 years, 7,512 received a screening urinary test (RAPIRAN ™; Otsuka Pharmaceutical Co., Ltd., Tokyo, Japan) to detect anti-*H. pylori* immunoglobulin-G antibody by immunochromatography. A screening program for kidney diseases was established in Saga Prefecture, which targets third-grade students in junior high school. Given the full inclusivity of students during this test through simple urine examination, the established system was used to obtain urine samples to screen for *H. pylori* infection. A total of 7,325 students tested negative for *H. pylori* with the urinary test. Among 187 students who were tested positive in the screening urinary test underwent an *H. pylori* stool antigen test (SAT) (Quick Chaser ™ *H. pylori* [Q (IC)]; Mizuho Medy Co., Ltd., Tosu City, Saga, Japan). The following are the exclusion criteria for this study: (i) students who had taken medications, including proton pump inhibitors (PPIs), H2 receptor antagonists, antacids, probiotics, mucosal protective agents, and antibiotics within 6 months before enrollment; (ii) students who had outpatient hospital visits because of sickness; (iii) students with chronic gastrointestinal diseases, such as functional gastrointestinal disorders, celiac disease, eosinophilic gastrointestinal disorder, inflammatory bowel diseases, etc.; and (iv) students who had undergone eradication therapy for *H. pylori*.

### 2.3. B (EIA) vs. Q (IC)

A fecal *H. pylori* antigen test was performed in 141 students as a secondary screening using Q (IC) in our program. Q (IC) was measured following the methods described in the manufacturer's instructions. B (EIA) was measured with a fully automatic biochemical luminescence immunoassay device (BLEIA ™-1200) and a BL Sampling Bottle (Eiken Chemical Co., Ltd., Tokyo, Japan). The measurement was carried out by installing BL Sampling Bottle on the measuring device on which the reagent was already installed. The measurement data were obtained as a cutoff index (COI), which is a value obtained by dividing the amount of light emitted from each sample by the cutoff value calculated from the amount of light emitted by the calibrator. A COI of 1.0 or higher was considered positive and a COI of <1.0 was considered negative.

### 2.4. Comparison of Multiple Fecal Antigen Test Kits

A comparative test was conducted between B (EIA) and extracorporeal diagnostic agents that were marketed in Japan as of 2019. The comparison target tests were Q (IC), Testmate pylori antigen EIA (T[EIA]; Hitachi Kasei Diagnostics Systems Co., Ltd., Tokyo, Japan), Testmate rapid pylori antigen (T[IC]; Nippon Becton Dickinson Co., Ltd., Tokyo, Japan), Meridian HpSA ELISA II (M[EIA]; Pacific Bridge Medical Co., Ltd., Tokyo, Japan), and Immunocard STAT HpSA (I[IC]; Pacific Bridge Medical Co., Ltd., Tokyo, Japan). Measurements by Q (IC), T (EIA), T (IC), M (EIA), and I (IC) were performed according to the manufacturer's instructions. The equipment used for the measurement was BLEIA ™-1200 for B (EIA) based on the BLEIA method, and an immunochromatographic reader C10066 (Hamamatsu Photonics Co., Ltd., Hamamatsu, Shizuoka) for Q (IC), T (IC), and I (IC) based on the immunochromatography method. T (EIA) and M (EIA) based on the EIA method used plate readers Infinite 200 PRO M Plex (Tekan Japan Co., Ltd., Kawasaki, Kanagawa). In addition, the reagent based on the immunochromatography method was visually evaluated using the comprehensive determination of the immunochromatographic reader.

### 2.5. The Detection Performance of *H. pylori* ATCC43504 Standard Strain

The sample to confirm the detection performance was prepared by diluting 1.5 mg/mL of *H. pylori* antigen (ATCC43504 strain) with a buffer solution in a stool collection container that is dedicated to each reagent of 10–20,000 pg/mL.

### 2.6. The Detection Performance of *H. pylori* Antigen in Commercial Human Fecal Specimens

Commercially available *H. pylori* antigen-positive human feces (Discovery Life Sciences, Inc) were collected and suspended in stool collection containers that are dedicated to each test kit, and the obtained stool suspensions were diluted with the buffer solution of each company's stool collection containers. A total of 5 types of *H. pylori* antigen-positive human feces and 5 types of *H. pylori* antigen-negative human feces were prepared.

## 3. Results

### 3.1. B (EIA) vs. Q (IC)

**Figure 2:**
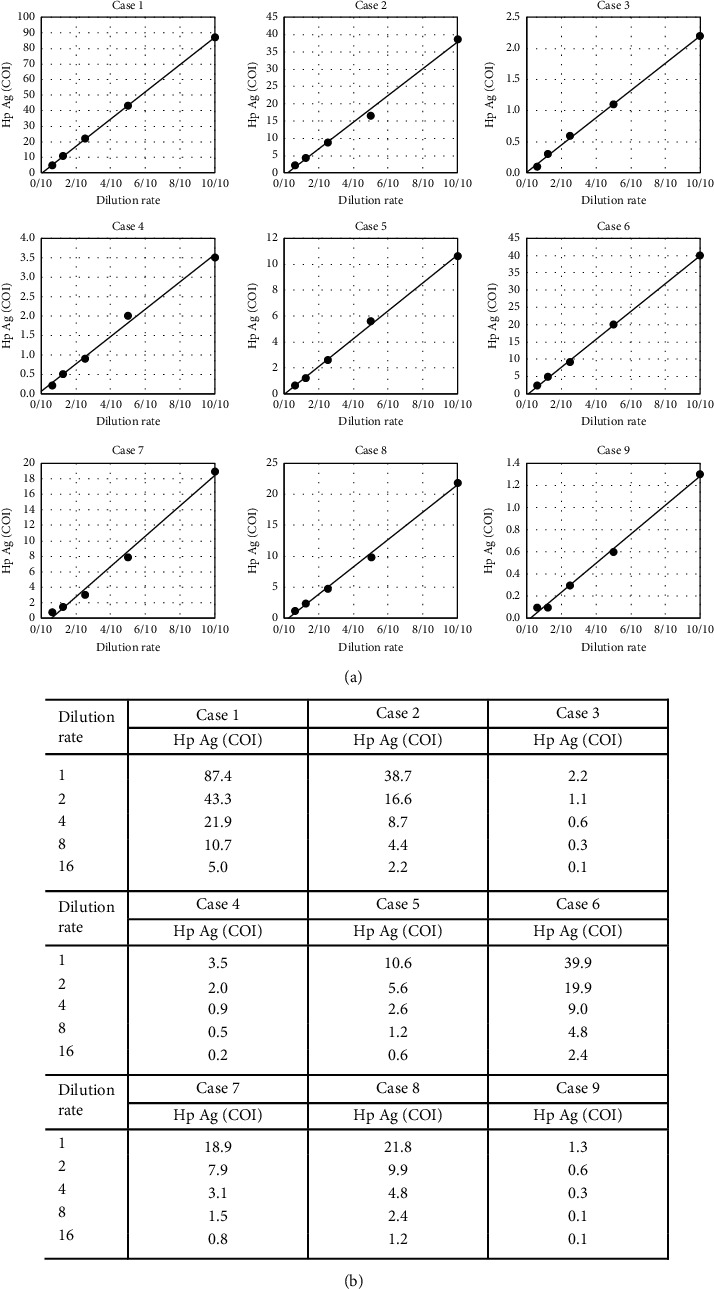
Dilution tests of 9 dissociated samples between B (EIA) and Q (IC). All samples almost showed linearity, without the characteristics seen in the nonspecific reaction (a). *Helicobacter pylori* antigen cutoff values for 9 samples without dilution ranged from 1.3 to 87.4 on (b) (EIA). Hp Ag, *Helicobacter pylori* antigen; COI, cutoff index.

Table 1 shows the correlation test results using Q (IC) and B (EIA) in 141 participants. The positive and negative concordance ratios of B (EIA) to Q (IC) were 100.0% (110/110) and 71.0% (22/31), respectively. The overall concordance ratio was 93.6% (132/141). A comparative study of B (EIA) and Q (IC) confirmed 9 divergent samples. A dilution linearity test was conducted in 9 cases to estimate the cause of result divergence. The dilution test sample of the dissociated sample was prepared by diluting the stool suspension that was collected and suspended in BL Sampling Bottle 2 to 16 times with the buffer solution of BL Sampling Bottle. As a result, all samples almost showed linearity, without the characteristics seen in the nonspecific reaction (Figure 2(a)). *H. pylori* antigen cutoff values for 9 samples without dilution ranged from 1.3 to 87.4 on B (EIA) (Figure 2(b)).

### 3.2. The Detection Performance of *H. pylori* Antigen of ATCC43504 Standard Strain

The measurement of *H. pylori* antigen dilution series of 10–20,000 pg/mL that was prepared by diluting 1.5 mg/mL of *H. pylori* antigen (ATCC43504 standard strain) resulted in positive ranges of B (EIA) as 39 pg/mL or more; Q (IC) as 2,500 pg/mL or more; T (EIA), T (IC), and M (EIA) as 10,000 pg/mL or more; and I (IC) as 20,000 pg/mL or more (Table 2).

### 3.3. The Detection Performance of *H. pylori* Antigen in Commercial Human Fecal Specimens

The measurement of the dilution series of the suspension of 5 commercially available *H. pylori* antigen-positive human fecal samples resulted in 128–3072 dilution times in B (EIA), 8–48 times in Q (IC), 4–48 times in T (EIA), 2–48 times in T (IC), 8–192 times in M (EIA), and 4–96 times in I (IC), which were positive (Table 3). The measurement of 5 commercially available *H. pylori* antigen-negative fecal samples was negative in all reagents (Supplementary Table 1).

## 4. Discussion

B (EIA) can be positive for *H. pylori* antigen of standard strain even in samples that are diluted for 64–512 times more than the other reagents and positive for *H. pylori* antigen in commercial human fecal specimens even in samples that are diluted for 16–128 times more than the other reagents. Therefore, B (EIA) is suggested to be the reagent with the highest detection performance of *H. pylori* antigen compared with other reagents.

A comparison between the newly developed B (EIA) and Q (IC) showed a positive concordance rate of 100%, the negative concordance rate of 71.0%, and overall concordance rate of 93.6%, which was considered a favorable result. Nine dissociated specimens that were negative for Q (IC) and positive for B (EIA) were confirmed. All dilution tests of these 9 cases almost showed linearity, without characteristics of nonspecific reactions. Additionally, the measured value of B (EIA) of the dissociation samples was 1.3–87.4 COI (Figure 2(a)) in the range that can be evaluated as negative by other fecal *H. pylori* antigen test kits (Table 2); thus, all dissociation samples were *H. pylori* antigen-positive cases, and finally the cause of the result divergence was presumed to be false negative due to insufficient Q (IC) sensitivity.

The guidelines for *H. pylori* infection management in Japan suggested that the SAT has great diagnostic performance, with a sensitivity of 96%–100% and specificity of 97%–100% before eradication [21]. Diagnostic performances of different SATs are heterogeneous, which may relate to the designs of tests like EIA and immunochromatographic assay (ICA) and for the selection of antibodies, such as monoclonal and polyclonal antibodies [22]. Many studies were conducted on the performance evaluations of SAT kits [23–27], but this is the first study that simultaneously evaluated six types of SAT kits. EIA provides more reliable results than ICA [28]. In the present study, B (EIA) was considered to have better sensitivity than the EIA and IC method for *H. pylori* antigen.

In Japan, the number of municipalities conducting screening for *H. pylori* among junior high school students has increased in recent years to prevent gastric cancer [12–14, 29, 30]. *H. pylori* antigen testing using fecal samples is adopted because of its simplicity in screening. Moreover, it is an extremely useful examination method because of its conduction without endoscopy, and such a method is preferred in children. Attempts to extract the DNA of *H. pylori* bacteria and test resistance to *H. pylori* against clarithromycin using stool samples have been assessed [31, 32]. In the methods using feces, the fecal antigen and drug susceptibility tests can be performed using the same specimen, and the usefulness of the fecal antigen test will be further enhanced compared to UBT test. Also in this point, B (EIA) is a useful screening method to test and treat *H. pylori* in adolescents.

*H. pylori* was recognized as a pathogenic infectious agent for humans only in 1985 by Marshall and Warren [33]. The recognition of the pathogenic role of *H. pylori* has revolutionized medicine and gastric and duodenal pathology. This has led not only to the new description of the etiopathogenesis of some diseases of the digestive system, but also to re-thinking the prevention and therapy; for example, acute gastritis, chronic atrophic gastritis, ulcer of stomach and duodenum, mucosa-associated lymphoid tissue lymphoma, esophageal cancer, gastric adenocarcinoma, nonalcoholic steatohepatitis, etc [34]. Based on these facts, it is also the basis for test and treatment to eradicate *H. pylori* during adolescence to prevent gastric cancer [13, 14].

It is well established that *H. pylori* infection is acquired in childhood [35]. Regarding eradication therapy among children from the perspective of future gastric cancer prevention, this point remains controversial. Therefore, we still need to discover the relationships between individual and environmental factors, including incidence and pathogenesis and correlations with other systems, in order to determine when to eradicate *H. pylori*. At present, there is a significant reduction in the incidence of the disease worldwide and a steady decrease in the incidence of infection in childhood, probably due to better conditions of hygiene and improvement in treatment. Treatment must address compliance and antibiotic resistance during the disease [36, 37]. In Japan, cases of eradication treatment failure largely depend on the presence or absence of clarithromycin resistance [38], with CAM-resistant rate being higher in children [39]. Several studies have confirmed that probiotic supplementation is able to facilitate eradication and reduce the incidence of side effects of antibiotic therapy [34], because the microbiota can be altered by immune-mediated reactions of the organism against the bacterium [40].

The present research has limitations that need to be addressed in future studies. Study results were not compared to the gold standard urea breath test or invasive tests (bacterial culture, rapid urease test, etc.), thus positive or negative results were still controversial. The present study revealed that B (EIA) had a beneficial result in *H. pylori* infection diagnosis. However, research on the eradication therapy efficacy has not been conducted. Furthermore, the effect of oral antacid administration, such as PPI, has not been evaluated. The impact of PPI is believed to be minimal using the fecal antigen test kit [41, 42]; however, this assumption was not validated. The evaluation due to the differences in the genotype of *H. pylori* was not eventually performed using B (EIA).

## 5. Conclusions

B (EIA) is based on the BLEIA method that applies firefly luciferase luminescence and is more sensitive than the SAT kits that are currently marketed in Japan. This test is very useful for diagnosing *H. pylori* infections, particularly in cases where noninvasive tests are preferred, such as in children.

## Figures and Tables

**Table 1:** Concordance rate of the evaluation reagent “B (EIA]” for the target reagent “Q (IC).”

	B (EIA)	
	Positive (*n* = 119)	Negative (*n* = 22)
Q (IC)		
Positive (*n* = 110)	110	0
Negative (*n* = 31)	9	22

Q (IC), Quick Chaser™ *H. pylori*; B (EIA), BLEIA™ “EIKEN” *H. pylori* antigen.

**Table 2:** Detection of *H. pylori* antigen of ATCC43504 standard strain.

Hp Ag (pg/mL)	Measured values						Judgement					
	B (EIA)	Q (IC)	T (EIA)	T (IC)	M (EIA)	I (IC)	B (EIA)	Q (IC)	T (EIA)	T (IC)	M (EIA)	I (IC)
	(COI)	(mAbs)	(AbS)	(mAbs)	(Abs)	(mAbs)						
20,000	644.2	31.7	0.283	20.9	0.343	9.8	＋	＋	＋	＋	＋	＋
10,000	396.5	22.9	0.161	8.7	0.161	1.3	＋	＋	＋	＋	＋	−
5,000	166.2	13.2	0.089	3	0.052	0	＋	＋	−	−	−	−
2,500	91.9	8.6	0.079	1.8	0.009	0	＋	＋	−	−	−	−
1,250	45.5	0	0.044	0	0.006	1.5	＋	−	−	−	−	−
625	21.8	0	0.034	0	0.005	0	＋	−	−	−	−	−
313	11.4	0	0.034	0	0.005	0	＋	−	−	−	−	−
156	5.5	0	0.023	0	0.004	0	＋	−	−	−	−	−
78	2.3	0	0.023	0	0.01	0	＋	−	−	−	−	−
39	1.2	0	0.023	0	0.002	0	＋	−	−	−	−	−
20	0.5	0	0.029	0	0.002	0	−	−	−	−	−	−
10	0.3	0	0.023	0	0.002	0	−	−	−	−	−	−
Diluted solution	0	0	0.023	0	0.004	0	−	−	−	−	−	−

Hp Ag, *Helicobacter pylori* antigen; B (EIA), BLEIA ™ “EIKEN” *H. pylori* antigen; Q (IC), Quick Chaser ™ *H. pylori*; T (EIA), Testmate pylori antigen EIA; T (IC), Testmate rapid pylori antigen; M (EIA), Meridian HpSA ELISA II; I (IC), Immunocard STAT HpSA.

**Table 3:** Detection of *H. pylori* antigen in commercial human fecal specimens.

Sample	Dilution rate	Measured values						Judgement					
		B (EIA)	Q (IC)	T (EIA)	T (IC)	M (EIA)	I (IC)	B (EIA)	Q (IC)	T (EIA)	T (IC)	M (EIA)	I (IC)
		(COI)	(mAbs)	(AbS)	(mAbs)	(Abs)	(mAbs)						
Specimen 1	×24	166.4	29.2	0.243	14.5	1.143	8.6	＋	＋	＋	＋	＋	＋
	×48	87	17.7	0.14	5.2	0.631	2.4	＋	＋	＋	＋	＋	＋
	×96	38.5	0	0.078	2.9	0.284	2.8	＋	−	−	−	＋	＋
	×192	21.6	0	0.051	0	0.106	0	＋	−	−	−	＋	－
	×384	10.6	0	0.034	0	0.015	0	＋	−	−	−	−	−
	×768	5.3	0	0.029	0	0.003	0	＋	−	−	−	−	−
	×1536	2.7	0	0.025	0	0.006	0	＋	−	−	−	−	−
	×3072	1.4	0	0.021	0	0.004	0	＋	−	−	−	−	−
	×6144	0.7	0	0.02	0	0.006	0	−	−	−	−	−	−

Specimen 2	×12	167.5	28	0.221	9.2	1.985	6	＋	＋	＋	＋	＋	＋
	×24	81.5	17.2	0.12	0	0.979	5.7	＋	＋	＋	−	＋	＋
	×48	42.3	8.9	0.07	1.5	0.364	0	＋	＋	−	−	＋	−
	×96	20.1	0	0.046	0	0.125	0	＋	−	−	−	＋	−
	×192	10	0	0.033	0	0.027	0	＋	−	−	−	−	−
	×384	5.7	0	0.027	0	0.007	0	＋	−	−	−	−	−
	×768	2.6	0	0.025	0	0.005	0	＋	−	−	−	−	−
	×1536	1.3	0	0.024	0	0.007	0	＋	−	−	−	−	−
	×3072	0.7	0	0.024	0	0.005	0	−	−	−	−	−	−

Specimen 3	×12	130.4	21.6	0.159	6.5	0.568	6.5	＋	＋	＋	＋	＋	＋
	×24	65.9	13.3	0.09	4.8	0.234	2.5	＋	＋	−	−	＋	＋
	×48	31.3	5	0.056	0	0.089	0	＋	＋	−	−	−	−
	×96	17.1	0	0.038	0	0.017	0	＋	−	−	−	−	−
	×192	8.1	0	0.03	0	0.008	0	＋	−	−	−	−	−
	×384	4.2	0	0.026	0	0.005	0	＋	−	−	−	−	−
	×768	2.2	0	0.025	0	0.005	0	＋	−	−	−	−	−
	×1536	1.1	0	0.021	0	0.004	0	＋	−	−	−	−	−
	×3072	0.6	0	0.022	0	0.006	0	−	−	−	−	−	−

Specimen 4	×2	112.7	20.3	0.157	5.1	0.386	8.6	＋	＋	＋	＋	＋	＋
	×4	53.2	12	0.101	3.5	0.205	5.2	＋	＋	＋	−	＋	＋
	×8	27.4	7.3	0.059	2.8	0.139	0	＋	＋	−	−	＋	−
	×16	13.8	0	0.039	0	0.071	0	＋	−	−	−	−	−
	×32	6.9	0	0.028	0	0.01	0	＋	−	−	−	−	−
	×64	3.4	0	0.025	0	0.004	0	＋	−	−	−	−	−
	×128	1.8	0	0.026	0	0.006	0	＋	−	−	−	−	−
	×256	0.9	0	0.023	0	0.004	0	−	−	−	−	−	−
	×512	0.5	0	0.021	0	0.007	0	−	−	−	−	−	−

Specimen 5	×4	124.4	17.4	0.17	17.4	1.438	11.4	＋	＋	＋	＋	＋	＋
	×8	62.8	7.6	0.099	6.5	0.622	11.6	＋	＋	−	＋	＋	＋
	×16	30.5	0	0.057	0	0.233	6.4	＋	−	−	−	＋	＋
	×32	15.3	0	0.038	1.1	0.069	0	＋	−	−	−	−	−
	×64	7.7	0	0.029	0	0.011	0	＋	−	−	−	−	−
	×128	4	0	0.026	0	0.005	0	＋	−	−	−	−	−
	×256	2	0	0.022	0	0.005	0	＋	−	−	−	−	−
	×512	1	0	0.022	0	0.005	0	＋	−	−	−	−	−
	×1024	0.5	0	0.022	0	0.003	0	−	−	−	−	−	−

B (EIA), BLEIA ™ “EIKEN” *H. pylori* antigen; Q (IC), Quick Chaser ™ *H. pylori*; T (EIA), Testmate pylori antigen EIA; T (IC), Testmate rapid pylori antigen; M (EIA), Meridian HpSA ELISA II; I (IC), Immunocard STAT HpSA.

## Data Availability

The datasets used and analyzed during the current study are available from the corresponding author on reasonable request.

## Supplementary Materials

Supplementary Materials Supplementary Table 1: Detection of *H. pylori* antigen negative in commercial human fecal specimens.
