# Supplementary material for: Clinical Evaluation of a Novel Stool Antigen Test Using Bioluminescent Enzyme Immunoassay for Detecting Helicobacter pylori
Source: Can J Gastroenterol Hepatol. 2022 Apr 21;2022:5571542. doi: 10.1155/2022/5571542 (PMC9050312; doi:10.1155/2022/5571542)
Supplement: Supplementary Materials — Supplementary Table 1: Detection of H. pylori antigen negative in commercial human fecal specimens. [file 5571542.f1.docx]

**Supplementary Table 1** Detection of *H. pylori* antigen negative in commercial human fecal specimens

| Sample | Measured values | | | | | | Judgement | | | | | |
| --- | --- | --- | --- | --- | --- | --- | --- | --- | --- | --- | --- | --- |
|  | B(EIA) | Q(IC) | T(EIA) | T(IC) | M(EIA) | I(IC) | B(EIA) | Q(IC) | T(EIA) | T(IC) | M(EIA) | I(IC) |
|  | (COI) | (mAbs) | (AbS) | (mAbs) | (Abs) | (mAbs) |  |  |  |  |  |  |
| Specimen 6 | 0 | 0 | 0.017 | 0 | 0.004 | 0 | － | － | － | － | － | － |
| Specimen 7 | 0 | 0 | 0.015 | 0 | 0.005 | 0 | － | － | － | － | － | － |
| Specimen 8 | 0 | 0 | 0.011 | 0 | 0.006 | 1.2 | － | － | － | － | － | － |
| Specimen 9 | 0 | 0 | 0.021 | 3.8 | 0.005 | 0 | － | － | － | － | － | － |
| Specimen 10 | 0 | 0 | 0.017 | 0 | 0.005 | 0 | － | － | － | － | － | － |

B(EIA), BLEIA ^TM^ “EIKEN” *H. pylori* antigen

Q(IC), Quick Chaser ^TM^ *H. pylori*

T(EIA), Testmate pylori antigen EIA

T(IC), Testmate rapid pylori antigen

M(EIA), Meridian HpSA ELISA II

I(IC), Immunocard STAT HpSA
